# Supplementary material for: Halftone-encoded 4D printing of stimulus-reconfigurable binary domains for cephalopod-inspired synthetic smart skins
Source: Nat Commun. 2025 Nov 12;16:9931. doi: 10.1038/s41467-025-65378-8 (PMC12612155; doi:10.1038/s41467-025-65378-8)
Supplement: Supplementary file 1 — Supplementary information [file 41467_2025_65378_MOESM1_ESM.pdf]

Supplementary Information

Halftone-Encoded 4D Printing of Stimulus-Reconfigurable Binary Domains for Cephalopod-Inspired Synthetic Smart Skins

Haoqing Yang[1], Haotian Li[1], Juchen Zhang[1], Tengxiao Liu[2], H. Jerry Qi[3]*, and Hongtao Sun[1,4]*

*Corresponding author. Email: qih@me.gatech.edu; hongtao.sun@psu.edu

This file includes:

Supplementary Figs. 1-15

Supplementary Table 1

Other Supplementary Materials for this manuscript include the following:

Supplementary Movies 1-4 (.mp4)

Supplementary Figures

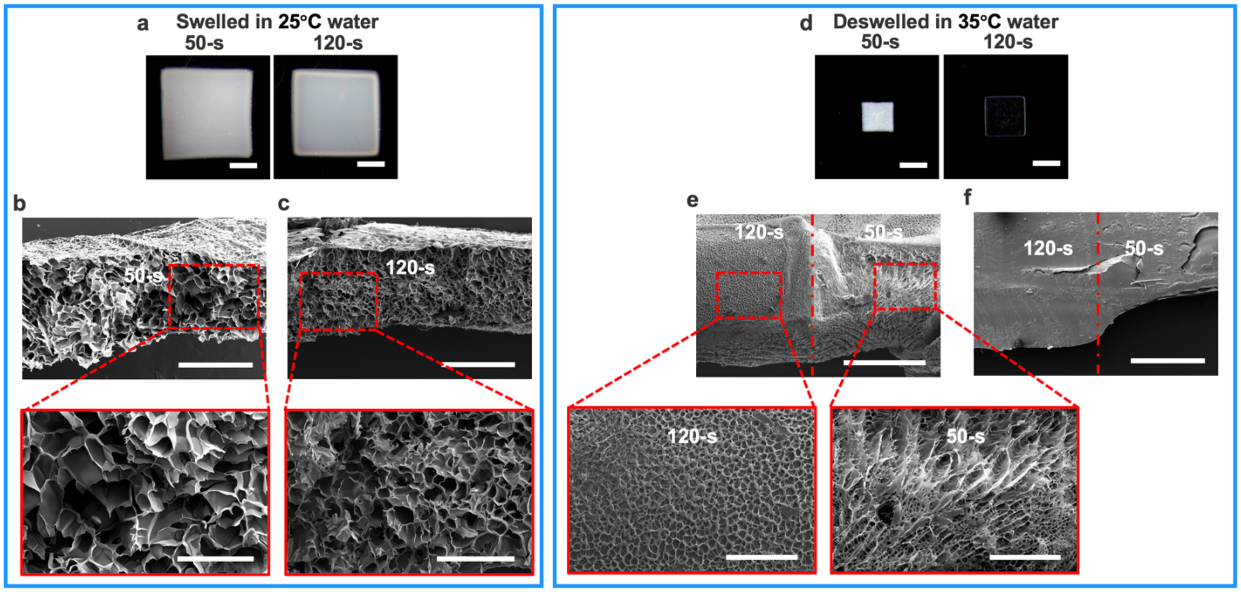

Supplementary Fig. 1. Optical appearance and morphological characterizations of hydrogel films under different swelling-deswelling states and solvent conditions. a, d, Optical appearance of hydrogel films printed with low (50-s, "0") and high (120-s, "1") UV exposures in the swollen state at 25 °C water (**a**), and in the deswollen state at 35 °C water (**d**) when placed against a black background. **b, c,** Cross-sectional views of swollen hydrogel films exposed to 50-s (**b**) and 120-s (**c**) UV curing. **e,** Top views of deswollen hydrogel films, illustrating distinct surface morphologies in the 50-s and 120-s regions. **f,** Cross-sectional view of deswollen hydrogel films with highly cross-linked (120-s) and lightly cross-linked (50-s) regions. Hydrogel films were freeze-dried prior to morphological characterizations. Scale bars: 5 mm (**a, d**); 500 µm (**b, c, f**); 400 µm (**e**); 200 µm, zoomed-in images in (**b, c**), 100 µm, zoomed-in images in (**e**).

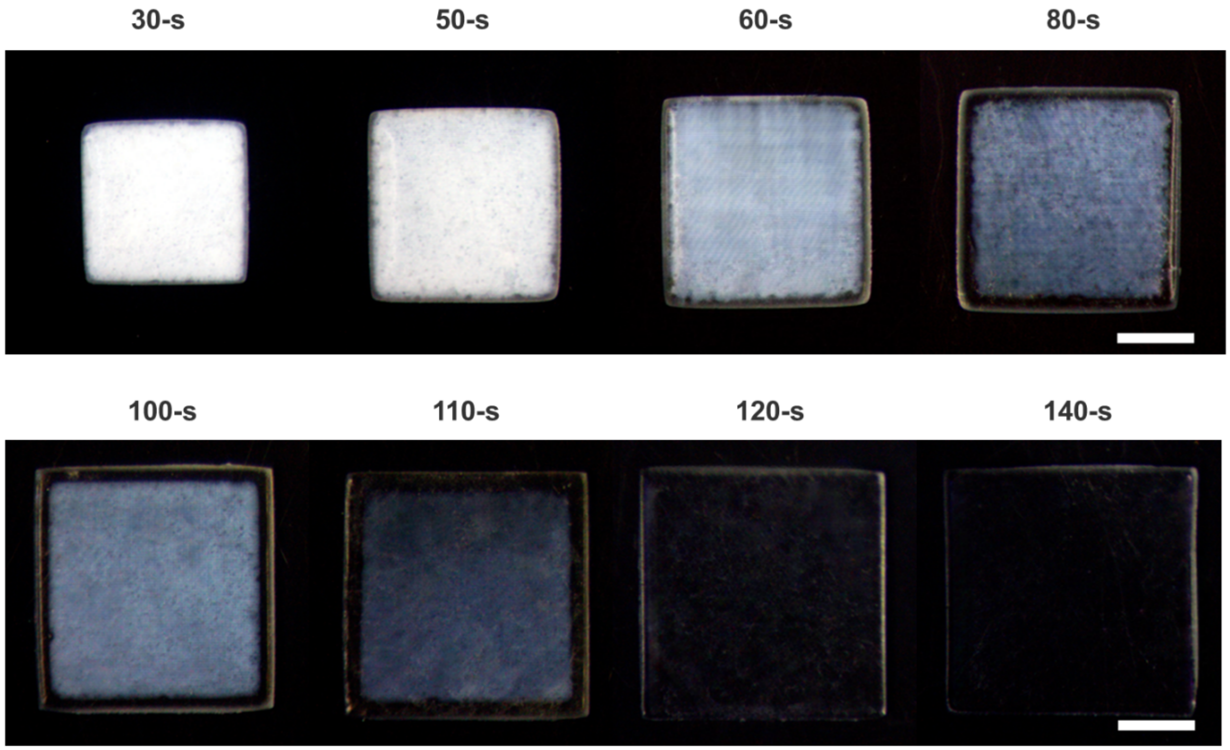

Supplementary Fig. 2. UV exposure-regulated optical appearance and deswelling-induced deformation. Optical views of deswollen hydrogel films regulated by UV exposures demonstrate a decrease in opacity with increased curing times (from 30 to 140 s) against a black background. Scale bars: 2 mm.

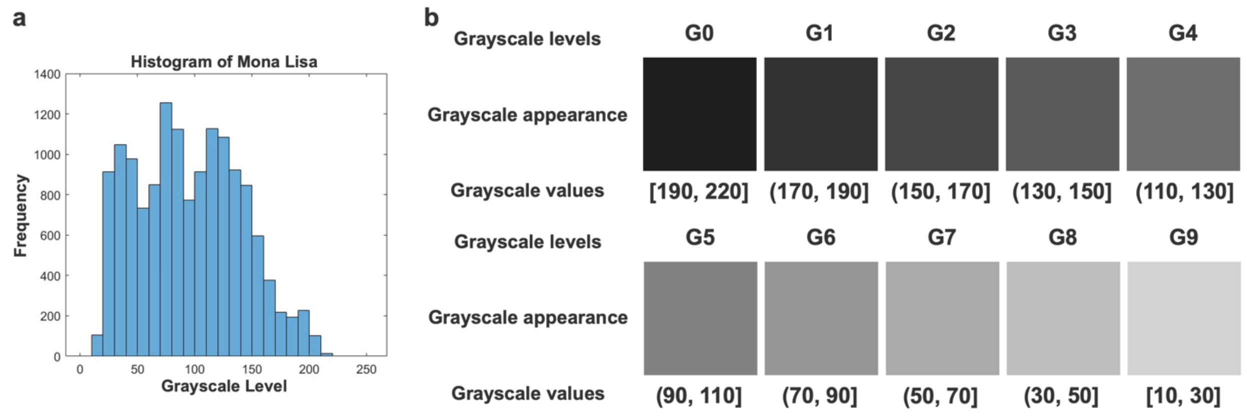

Supplementary Fig. 3. Process of creating an intermittent grayscale version to match defined grayscale levels generated by halftone methods. a, Frequency histogram of grayscale values in 120 × 120-pixel, 256-grayscale version of the Mona Lisa portrayal in Fig. 3d, ranging from 10 to 220. **b,** 256-grayscale levels are uniformly assigned into 10 intermittent bins, representing grayscale levels that match those generated by the defined halftone patterns from G0 to G9 (*e.g.*, Fig. 3a, b).

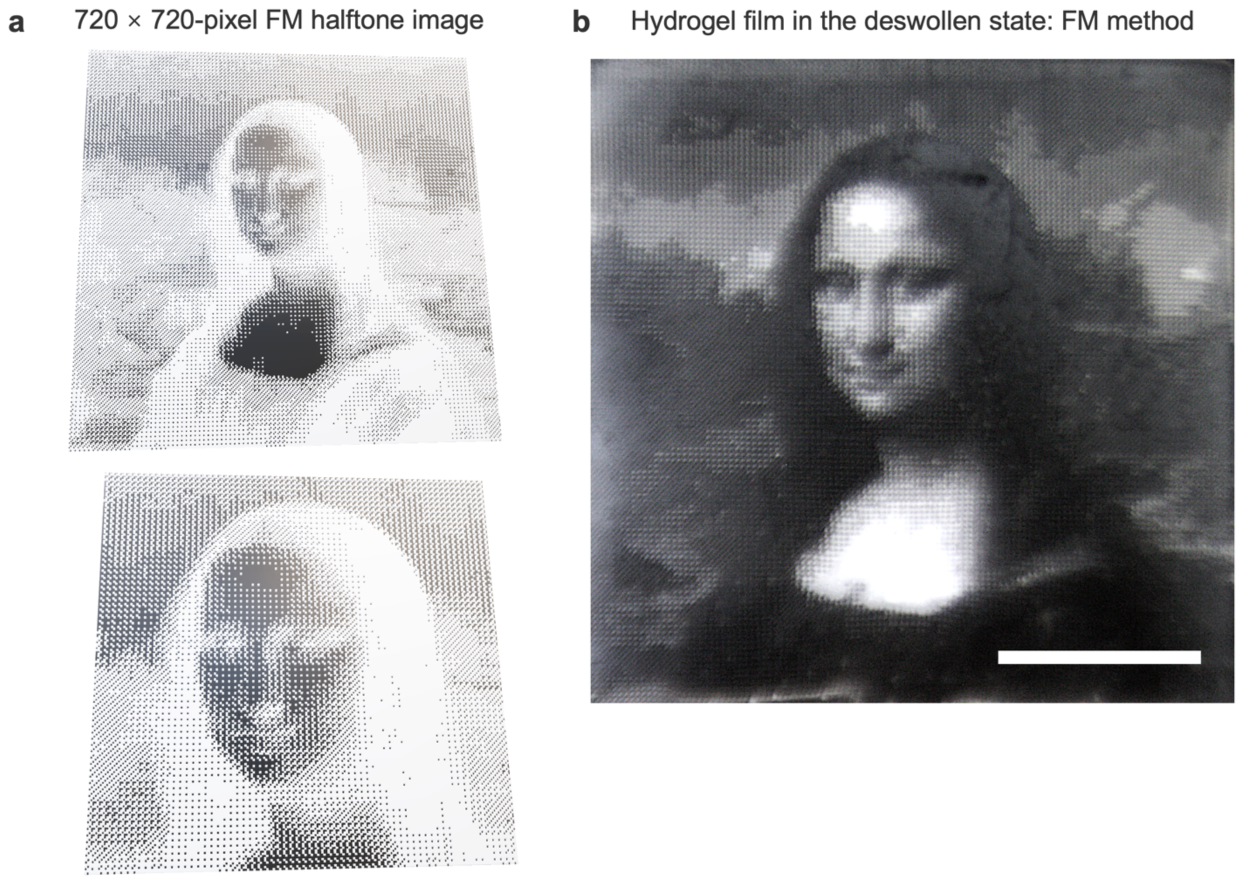

Supplementary Fig. 4. Halftone design using the FM method and a high-definition image of a hydrogel film displaying the Mona Lisa portrayal. a, A 720 × 720-pixel FM halftone image, where the varying heights of black and white pixels in the halftone design correspond to the controlled exposure levels (*e.g.*, black pixels with lower heights represent 50-second exposure, while white pixels with higher height represent 120-second exposure). **b,** The hydrogel film encoded with FM-generated halftone patterns briefly displays a white-and-black contrast when placed on a black background in the deswollen state at 35 °C. The resulting appearance reveals an inverse white-to-black visual relationship with the designed halftone patterns. Scale bar: 5 mm.

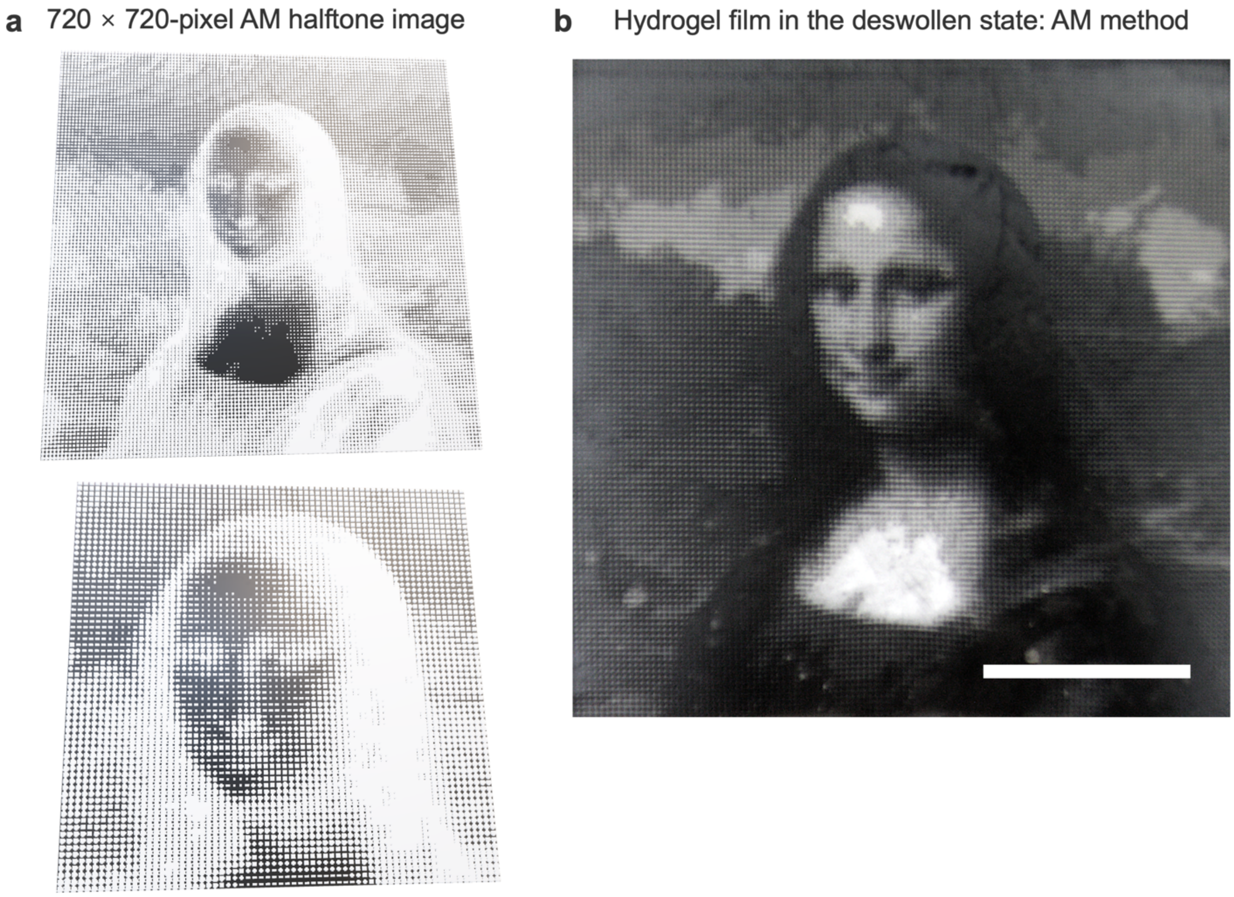

Supplementary Fig. 5. Halftone design using the AM method and a high-definition image of a hydrogel film displaying the Mona Lisa portrayal. a, A 720 × 720-pixel AM halftone image. **b,** Hydrogel film encoded with AM-generated halftone patterns in the deswollen state at 35 °C. Scale bar: 5mm. Note: The halftone design model incorporates two distinct heights, representing exposure levels of 50 seconds for black pixels with low height and 120 seconds for white pixels with high height.

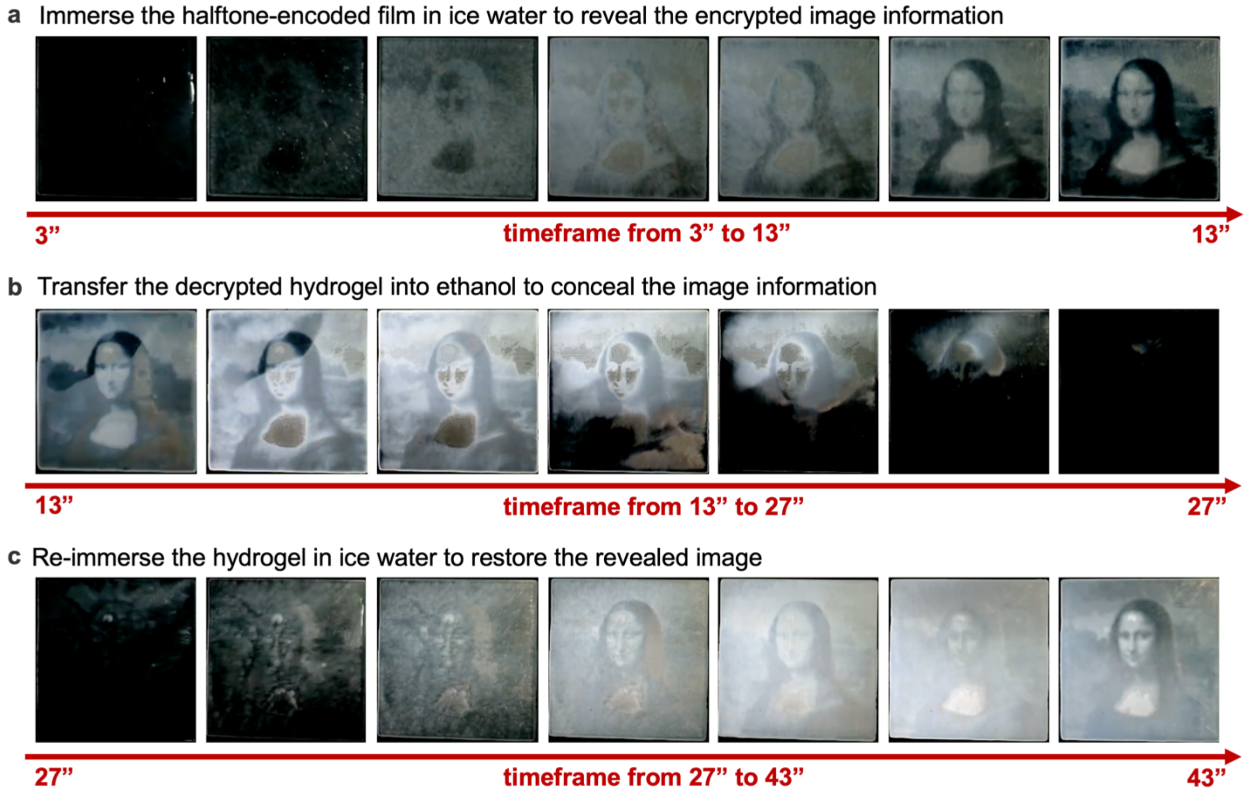

Supplementary Fig. 6. A halftone-encoded hydrogel film for repeatable and reversible image concealment and retrieval through an ethanol-water immersion cycle enabling dynamic information decryption. a, Timeframe 3”-13’’ from Supplementary Video 1: immerse the halftone-encoded hydrogel film in ice water to reveal the encrypted image information. **b,** Timeframe 13”-27” from Supplementary Video 1: transfer the decrypted hydrogel into ethanol to conceal the image information. **c,** Timeframe 27”-43” from Supplementary Video 1: re-immerse the hydrogel in ice water to restore the revealed image. Notes: the movie is played at 10 times the real-time speed.

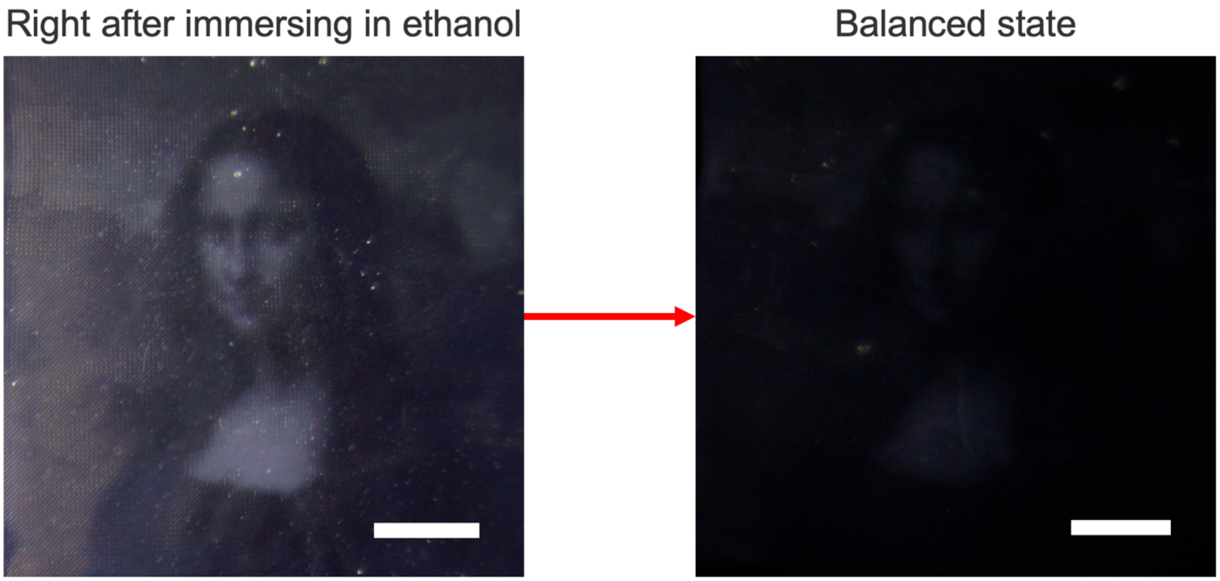

Supplementary Fig. 7. Rapid erasure of optical information in hydrogel films. The hydrogel film encoded with the Mona Lisa portrayal transitions to a transparent state upon immersion in ethanol at 35 °C. Scale bars, 5 mm.

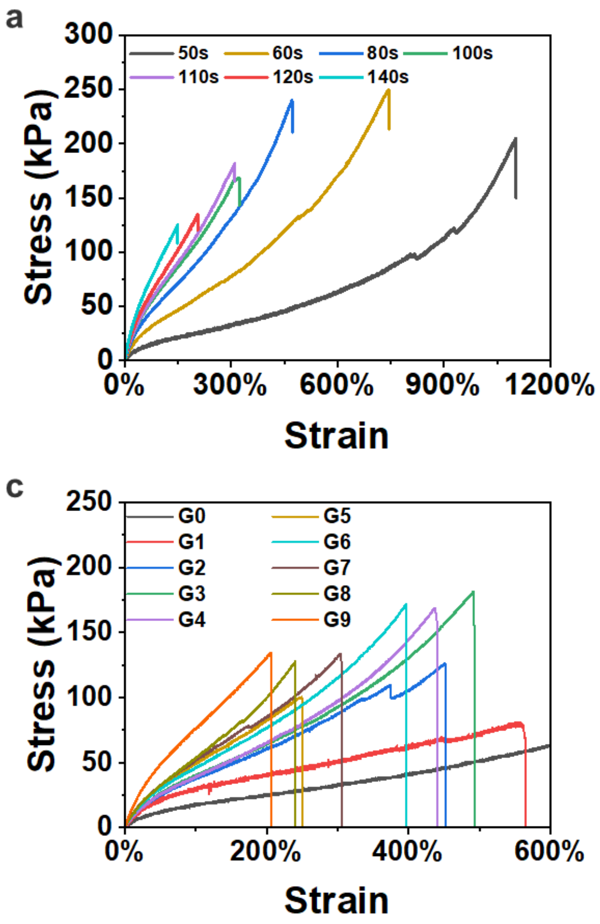

Supplementary Fig. 8. Mechanical characteristics of hydrogel films. a, Stress-strain curves of homogeneous hydrogel films exposed to varying UV does (*e.g.*, 50 seconds to 140 seconds). **b-d,** Stress-strain curves of hydrogel films encoded with FM (**b**) and AM (**c**) halftone patterns, along with a comparison of their Young's moduli (**d**).

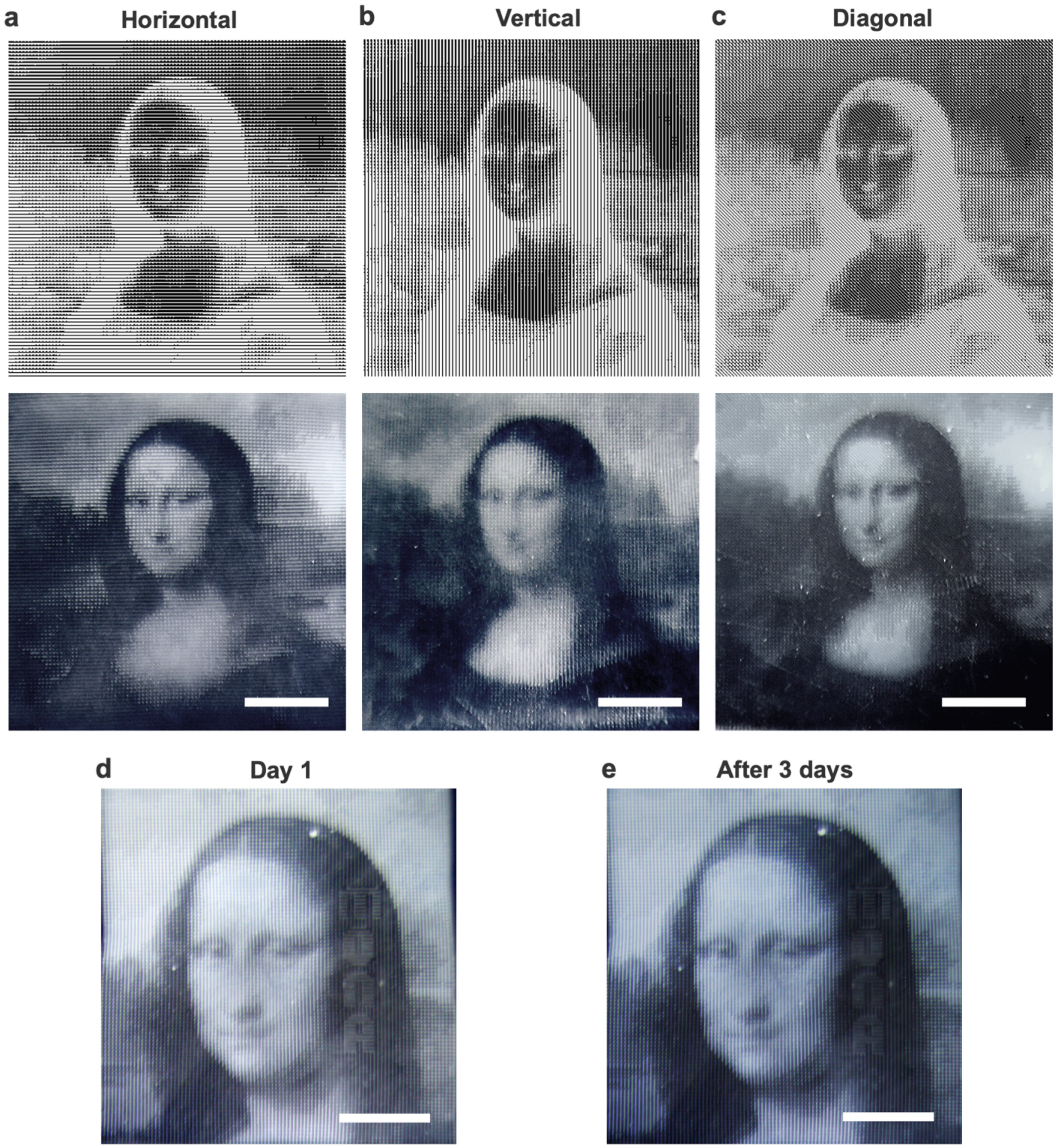

Supplementary Fig. 9. Hydrogel films encoded with oriented halftone patterns depicting the Mona Lisa. a-c, The halftone patterns are oriented horizontally (**a**), vertically (**b**), or diagonally (**c**) and span defined grayscale levels from G0 (16.7% "1" pixels per 6 × 6-pixel halftone unit matrix) to G9 (83.3% "1" pixels per 6 × 6-pixel unit matrix). **d,** Hydrogel film in the deswollen state on Day 1. **e,** Hydrogel film in the deswollen state after 3 days. Scale bars: 5 mm.

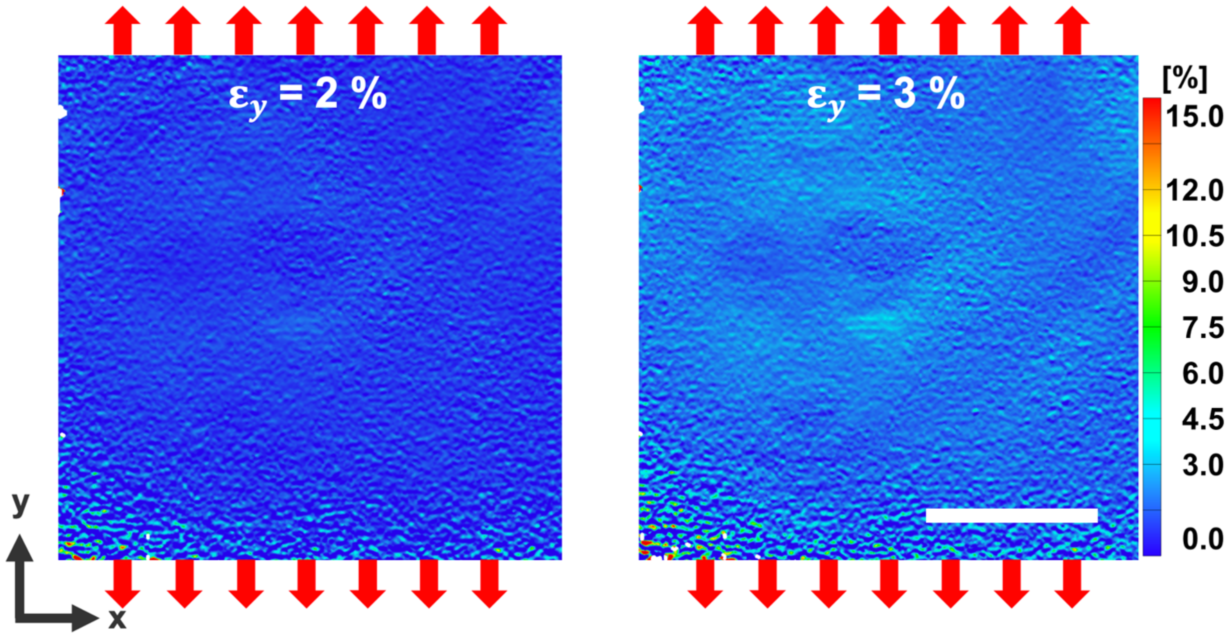

Supplementary Fig. 10. Full-field y-strain mapping of hydrogel films with vertically oriented halftone patterns under uniaxial stretching along the y-axis, as analyzed via DIC, which reveals less discernable features. Scale bar: 5 mm.

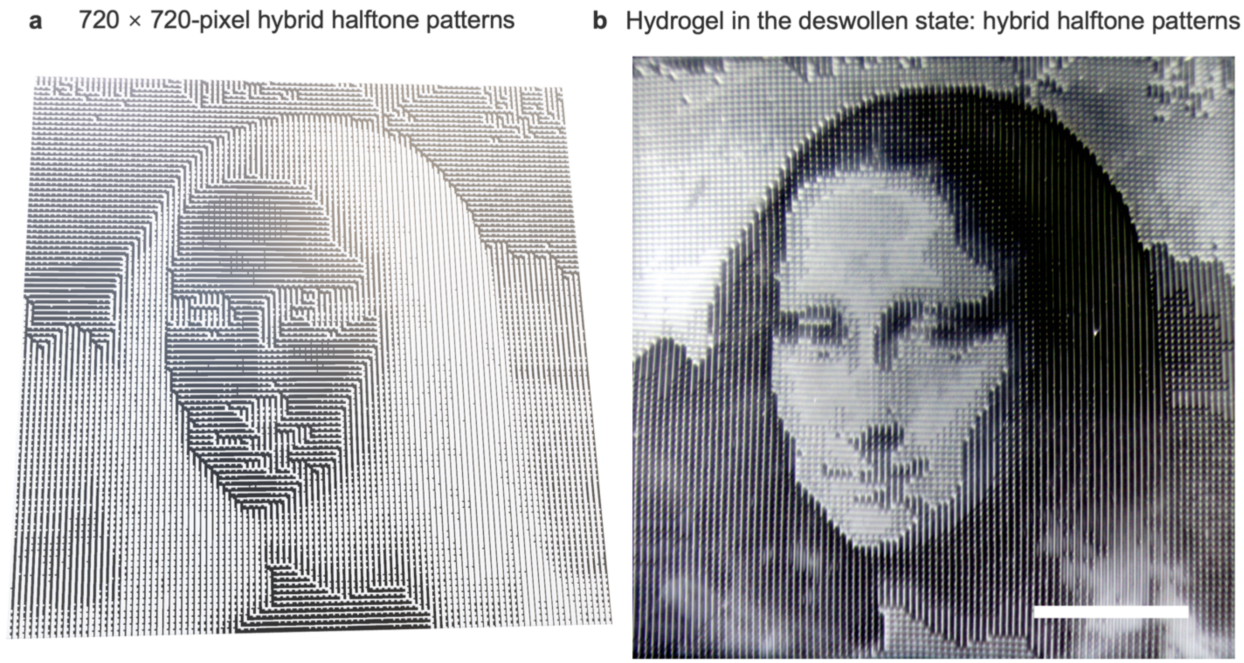

Supplementary Fig. 11. Hybrid halftone pattern design and high-definition image of a hydrogel film displaying the Mona Lisa. a, A 720 × 720-pixel hybrid halftone image, where darker or shadowed regions (*e.g.*, nose, eyes, mouth, facial shadows, and hairs) are selectively encoded with vertically arranged patterns at a same grayscale level, while other regions remain horizontally arranged patterns. **b,** Hydrogel film encoded with hybrid halftone patterns in the deswollen state at 35 °C. Scale bar: 5 mm.

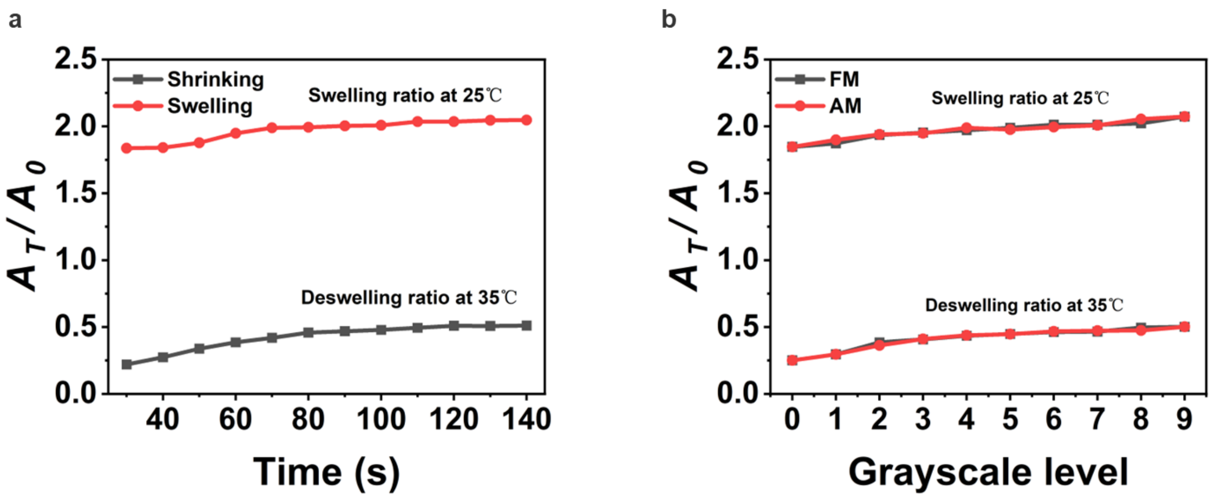

Supplementary Fig. 12. Swelling and deswelling ratios of thermoresponsive hydrogel films controlled by (**a**) varying curing times and (**b**) halftone-regulated binary patterns.

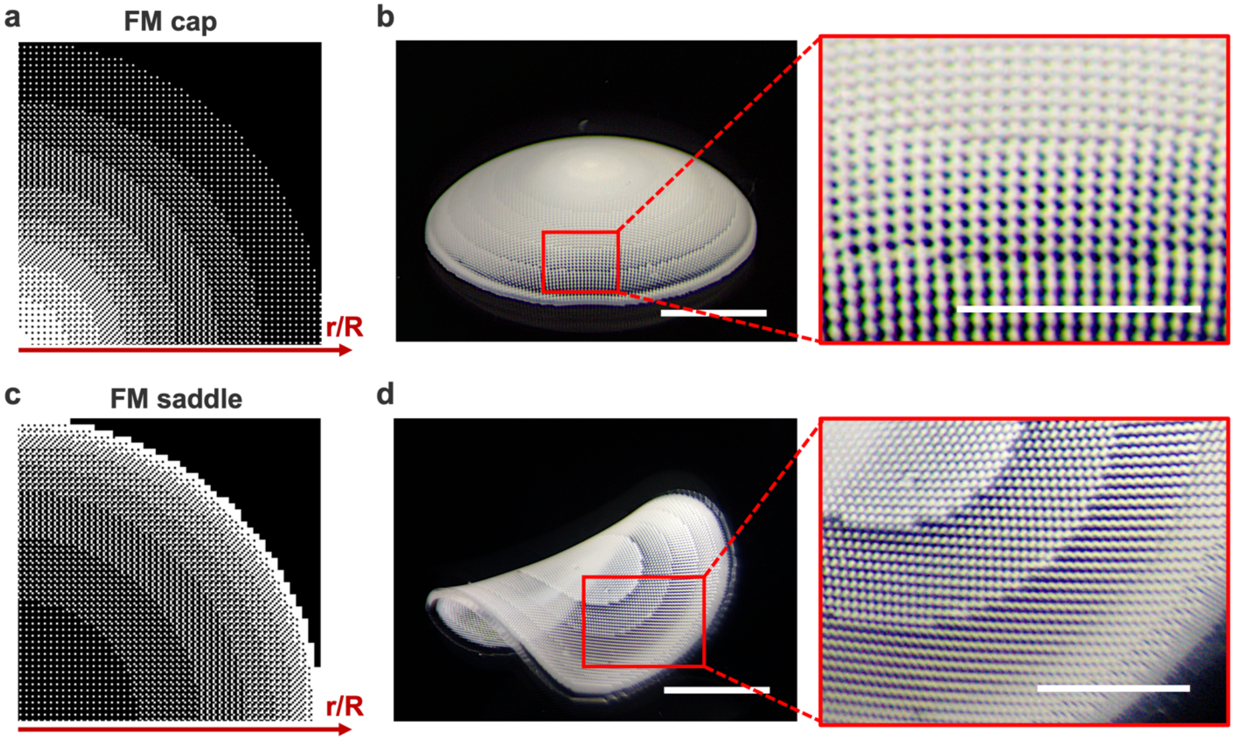

Supplementary Fig. 13. Halftone pattern-encoded growth functions enabling the transformation of 2D hydrogel films into axisymmetric non-Euclidean 3D shapes. a, b, Spatial design of halftone pattern-regulated grayscale gradients encoding a growth function $\boldsymbol{\eta} = \frac{\mathbf{A_{35℃}}}{\mathbf{A_0}} = \frac{c}{\left(1+\frac{r^2}{R}\right)^2}$, resulting in a 3D spherical cap after shape morphing. **c, d,** Spatial design of halftone pattern-regulated grayscale gradients encoding a growth function $\boldsymbol{\eta} = \frac{\mathbf{A_{35℃}}}{\mathbf{A_0}} = \frac{c}{\left(1-\frac{r^2}{R}\right)^2}$, resulting in a hyperbolic saddle after shape morphing. Here, *c* is constant, and *r/R* represents the relative radius of each concentric ring. An LCD-based 3D printer (Anycubic Photon Mono) was utilized to encode halftone pattern-regulated growth function in the hydrogels shown in this figure. These hydrogels exhibit distinct optical appearances compared to the 3D-morphed hydrogels printed with DLP 3D printer, due to differences in light sources and power. Scale bars: 5 mm (**b, d**), 2 mm (zoomed-in regions of **b, d**).

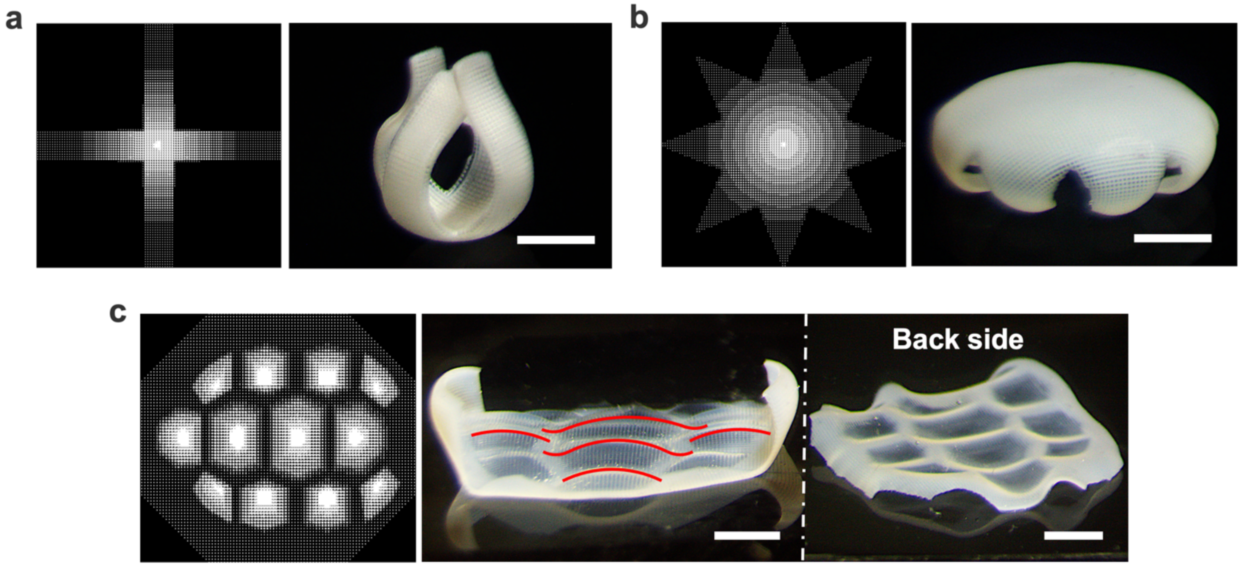

Supplementary Fig. 14. Halftone pattern-regulated transformation of 2D hydrogel films into various bioinspired 3D structures. a, b, Four-arm and eight-arm starfish shapes formed through halftone pattern-regulated shrinkage in the deswollen state. **c,** Complex surface morphologies achieved through 2D-to-3D shape morphing, mimicking bumpy "papillae" with controlled Gaussian curvatures in cephalopod skins. An LCD-based 3D printer (Anycubic Photon Mono) was utilized to encode halftone pattern-regulated growth function in the hydrogels demonstrated in this figure. Scale bars, 5 mm (**a-c**).

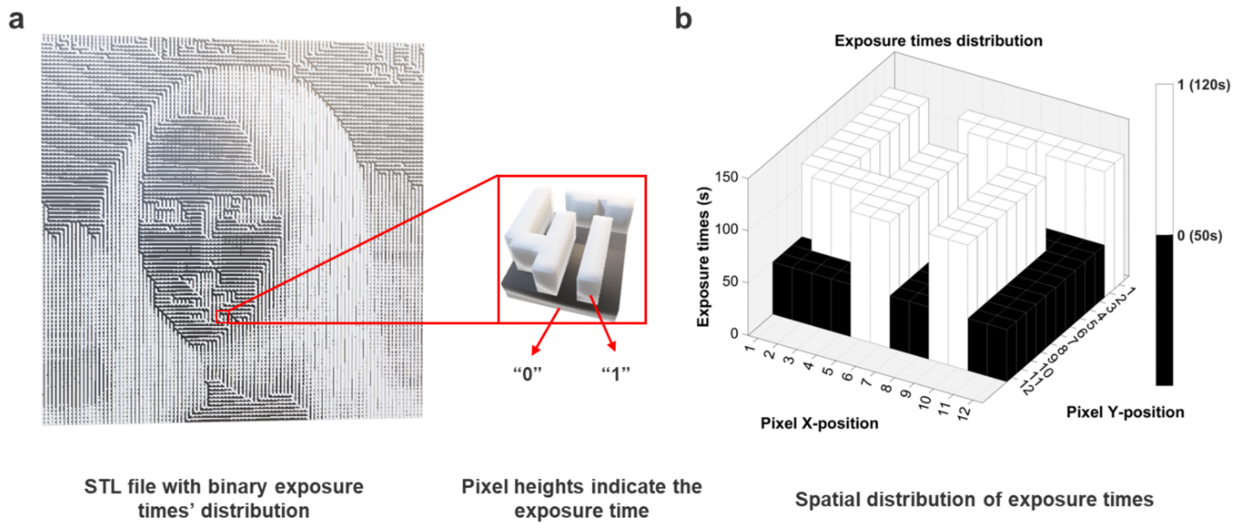

Supplementary Fig. 15. Halftone pattern-encoded model for fixed-height DLP printing. a, STL files of the halftone designs show varying pixel heights corresponding to controlled exposure levels: black pixels with lower heights represent 50-second exposure, whereas white pixels with greater heights represent 120-second exposure. **b,** Spatial distribution of exposure times illustrated by the different pixel heights in a zoomed-in region of (**a**).

Supplementary Tables

Supplementary Table 1. Summary of information encryption using smart materials.

Factors / Ref	Material Type	Decryption modalities	Decrypted image modes	Encryption-decryption mechanisms	Coloration
**Our work**	**Smart hydrogels**	**Optical/ Mechanical/ Optical-mechanical-dual mode**	**Continuous tones**	**Cononsolvency/ Transmission/ Mechanical heterogeneity/ Thermoresponsive deformations**	**Grayscale**
Ref 1	Gel composites	Optical	Fluorescent-responsive RGB colors	Chemical color	Polychrome
Ref 2	Quantum dot–polymer composites	Optical	Fluorescent-responsive RGB colors	Luminescent Perovskite Quantum Dot	Polychrome
Ref 3	Colloidal photonic crystals	Optical	RGB	Reflection/Diffraction	Polychrome
Ref 4	Shape memory polymers	Optical	Single tone	Scattering/Transmission	Monochrome
Ref 5	Polymers	Optical	Structural colors	Total internal reflection	Polychrome
Ref 6	Shape memory polymers	Optical	Fluorescent-responsive Blue colors	Chemical color	Monochrome
Ref 7	Elastomers	Optical/ Mechanical	Singles tone	Scattering	Monochrome
Ref 8	Shape memory polymers	Mechanical	Polarized polychrome	Birefringence	Polychrome
Ref 9	Liquid crystal elastomers	Optical	Continuous tones	Scattering/Transmission	Grayscale
Ref 10	Liquid crystals elastomers	Optical	Strain-responsive RGB colors	Reflection	Polychrome
Ref 11	Meta-structured hydrogels	Optical	Continuous tones	Scattering/Diffraction	Grayscale
Ref 12	Hydrogels	Optical/ Mechanical	Polarized polychrome	Birefringence	Polychrome
Ref 13	Fluorescence hydrogels	Optical	Fluorescent-responsive RGB colors	Chemical color	Green/Blue
Ref 14	Fluorescence hydrogels	Optical	Fluorescent-responsive Red colors	Chemical color	Red grayscale
Ref 15	LCST/UCST hydrogels	Optical	Singles tone	Transmission	Monochrome
Ref 16	Colloidal hydrogels	Optical	Singles tone	Transmission	Monochrome

Supplementary Table 1. Continued.

Factors / Ref	Information storage density	Temporal mode	Information stability	Information retention period
**Our work**	**µm**	**Static and Dynamic**	**Stable**	**Days**
Ref 1	mm	Static	Fluorescence quenching	Hours
Ref 2	mm	Static	Stable	Days
Ref 3	µm	Dynamic	Stable	N/A
Ref 4	N/A	Static	Need post-crosslinking	Minutes
Ref 5	µm	Static	Change with humidity	Minutes
Ref 6	mm	Static	Stable	N/A
Ref 7	µm	Dynamic	Stable	N/A
Ref 8	µm	Dynamic	Stable	N/A
Ref 9	µm	Dynamic	Stable	N/A
Ref 10	mm	Static and Dynamic	Stable	Permanent
Ref 11	µm	Dynamic	Stable	Permanent
Ref 12	mm	Dynamic	Nonstable	Hours
Ref 13	mm	Static	Fluorescence quenching	N/A
Ref 14	µm	Static	Stable	N/A
Ref 15	N/A	Static	Stable	N/A
Ref 16	N/A	Static	Stable	N/A

Supplementary Table 1. Continued.

Factors / Ref	Information read speed	Fidelity of information	Stimulus for information decryption	Multi-functionality
**Our work**	**Seconds/hours**	**High**	**Temperature/Stress /Solvent**	**2D-to-3D shape morphing**
Ref 1	N/A	Low	Solvent/UV light	Shape Bending
Ref 2	N/A	Low	UV light	N/A
Ref 3	Seconds	High	Temperature	Polychrome shape-shifting 3D structures
Ref 4	Minutes	High	Humidity	Shape memory 3D structures
Ref 5	Minutes	Medium	Humidity	N/A
Ref 6	Minutes	High	UV light	Shape memory 3D structures
Ref 7	Minutes	Low	Light/Temperature	N/A
Ref 8	Minutes	Medium	Stress	Stress-induced 2D-3D shapes
Ref 9	Seconds	High	Temperature/Stress	Biosensor
Ref 10	Seconds	High	Strain	N/A
Ref 11	Minutes	Relatively high	Temperature	3D shapes
Ref 12	Minutes	Low	Stress	Shape Bending
Ref 13	N/A	Medium	Metal Cation	N/A
Ref 14	Days	Medium	Temperature	Biocompatible, detection of metal ions
Ref 15	Minutes	Medium	Temperature	N/A
Ref 16	N/A	Medium	pH/Temperature	Strain senser

Note of N/A: This factor is not applicable

Supplementary References

(1) Sui, Y.; Li, C.; Feng, S.; Ling, Y.; Li, C.; Wu, X.; Shen, J.; Song, J.; Peng, H.; Huang, W. Patterning, morphing, and coding of gel composites by direct ink writing. *Journal of Materials Chemistry A* **2021**, *9* (13), 8586-8597. DOI: 10.1039/D0TA12275K.

(2) Jeon, H.; Wajahat, M.; Park, S.; Pyo, J.; Seol, S. K.; Kim, N.; Jeon, I.; Jung, I. D. 3D Printing of Luminescent Perovskite Quantum Dot–Polymer Architectures. *Advanced Functional Materials* **2024**, *34* (29), 2400594. DOI: https://doi.org/10.1002/adfm.202400594.

(3) Liao, J.; Ye, C.; Guo, J.; Garciamendez-Mijares, C. E.; Agrawal, P.; Kuang, X.; Japo, J. O.; Wang, Z.; Mu, X.; Li, W.; et al. 3D-printable colloidal photonic crystals. *Materials Today* **2022**, *56*, 29-41. DOI: https://doi.org/10.1016/j.mattod.2022.02.014.

(4) Liu, S.; Zhao, H.; Lyu, Y.; Wu, T.; Zhai, F.; Zhang, Y.; Ji, Z.; Wang, X. Grayscale stereolithography 3D printing of shape memory polymers for dual information encryption based on reconfigurable geometry and tunable optics. *Chemical Engineering Journal* **2024**, *487*, 150552. DOI: https://doi.org/10.1016/j.cej.2024.150552.

(5) Li, R.; Li, K.; Deng, X.; Jiang, C.; Li, A.; Xue, L.; Yuan, R.; Liu, Q.; Zhang, Z.; Li, H.; et al. Dynamic High-Capacity Structural-Color Encryption Via Inkjet Printing and Image Recognition. *Advanced Functional Materials* **2024**, *34* (42), 2404706. DOI: https://doi.org/10.1002/adfm.202404706.

(6) Huang, J.; Jiang, Y.; Chen, Q.; Xie, H.; Zhou, S. Bioinspired thermadapt shape-memory polymer with light-induced reversible fluorescence for rewritable 2D/3D-encoding information carriers. *Nature Communications* **2023**, *14* (1), 7131. DOI: 10.1038/s41467-023-42795-1.

(7) Zhang, L.; Jiang, X. Time-Dependent Wrinkle Pattern Based on Photo-Controlled Stress Relaxation for Multi-Level Information Encryption and Information Camouflage. *Advanced Functional Materials* **2024**, *34* (49), 2408932. DOI: https://doi.org/10.1002/adfm.202408932.

(8) Zhang, G.; Peng, W.; Wu, J.; Zhao, Q.; Xie, T. Digital coding of mechanical stress in a dynamic covalent shape memory polymer network. *Nature Communications* **2018**, *9* (1), 4002. DOI: 10.1038/s41467-018-06420-w.

(9) Choi, S. H.; Kim, J. H.; Ahn, J.; Kim, T.; Jung, Y.; Won, D.; Bang, J.; Pyun, K. R.; Jeong, S.; Kim, H.; et al. Phase patterning of liquid crystal elastomers by laser-induced dynamic crosslinking. *Nature Materials* **2024**, *23* (6), 834-843. DOI: 10.1038/s41563-024-01845-9.

(10) Kim, S.-U.; Lee, Y.-J.; Liu, J.; Kim, D. S.; Wang, H.; Yang, S. Broadband and pixelated camouflage in inflating chiral nematic liquid crystalline elastomers. *Nature Materials* **2022**, *21* (1), 41-46. DOI: 10.1038/s41563-021-01075-3.

(11) Zhang, M.; Pal, A.; Zheng, Z.; Gardi, G.; Yildiz, E.; Sitti, M. Hydrogel muscles powering reconfigurable micro-metastructures with wide-spectrum programmability. *Nature Materials* **2023**, *22* (10), 1243-1252. DOI: 10.1038/s41563-023-01649-3.

(12) Zhao, W.; Wu, B.; Lei, Z.; Wu, P. Hydrogels with Differentiated Hydrogen-Bonding Networks for Bioinspired Stress Response. *Angewandte Chemie International Edition* **2024**, *63* (21), e202400531. DOI: https://doi.org/10.1002/anie.202400531.

(13) Deng, J.; Wu, H.; Xie, W.; Jia, H.; Xia, Z.; Wang, H. Metal Cation-Responsive and Excitation-Dependent Nontraditional Multicolor Fluorescent Hydrogels for Multidimensional

Information Encryption. *ACS Applied Materials & Interfaces* **2021**, *13* (33), 39967-39975. DOI: 10.1021/acsami.1c12604.

(14) Wu, J.; Wang, Y.; Jiang, P.; Wang, X.; Jia, X.; Zhou, F. Multiple hydrogen-bonding induced nonconventional red fluorescence emission in hydrogels. *Nature Communications* **2024**, *15* (1), 3482. DOI: 10.1038/s41467-024-47880-7.

(15) Lou, D.; Sun, Y.; Li, J.; Zheng, Y.; Zhou, Z.; Yang, J.; Pan, C.; Zheng, Z.; Chen, X.; Liu, W. Double Lock Label Based on Thermosensitive Polymer Hydrogels for Information Camouflage and Multilevel Encryption. *Angewandte Chemie International Edition* **2022**, *61* (16), e202117066. DOI: https://doi.org/10.1002/anie.202117066.

(16) Zhu, C.; Zhang, L.; Zou, A.; Wang, W.; Zhang, J.; Zhang, A. A bionic intelligent hydrogel with multi-level information encryption and decryption capabilities. *Chemical Engineering Journal* **2023**, *475*, 146161. DOI: https://doi.org/10.1016/j.cej.2023.146161.
