# Supplementary material for: Halftone-encoded 4D printing of stimulus-reconfigurable binary domains for cephalopod-inspired synthetic smart skins
Source: Nat Commun. 2025 Nov 12;16:9931. doi: 10.1038/s41467-025-65378-8 (PMC12612155; doi:10.1038/s41467-025-65378-8)
Supplement: Supplementary file 2 — Description of Additional Supplementary Files [file 41467_2025_65378_MOESM2_ESM.pdf]

Description of Additional Supplementary Files

Supplementary Movie 1

Description: A halftone-encoded hydrogel film for repeatable and reversible image concealment and retrieval through an ethanol-water immersion cycle enabling dynamic information decryption. (1) Timeframe 3”-13’’: immerse the halftone-encoded hydrogel film in ice water to reveal the encrypted image information; (2) timeframe 13”-27”: transfer the decrypted hydrogel into ethanol to conceal the image information; (3) timeframe 27”-43”: re-immerse the hydrogel in ice water to restore the revealed image. Notes: the movie is played at 10 times the real-time speed.

Supplementary Movie 2

Description: A halftone-encoded hydrogel film dynamically reveals image information during the swelling-deswelling cycle as the temperature shifts between 25°C and 35°C, demonstrating its potential for information encryption-decryption. Notes: the movie is played at 500 times the real-time speed.

Supplementary Movie 3

Description: An FM halftone-encoded hydrogel film dynamically reveals graphic information under a small strain of 5%, captured using full-field strain mapping via real-time digital image correlation (DIC) analysis. Notes: the movie is played at 3 times the real-time speed.

Supplementary Movie 4

Description: After immersion in ethanol and water to erase the optical information, an FM halftone-encoded hydrogel film still preserves graphic information under a small strain, as decrypted using full-field strain mapping via real-time digital image correlation (DIC) analysis. Notes: the movie is played at 2 times the real-time speed.
